# Penta‐ and Hexacoordinated Copper(II) Complexes with Azido and 4‐amino‐3,5‐di‐2‐pyridyl‐4*H*‐1,2,4‐triazole Ligands with Field‐Induced Slow Magnetic Relaxation

**DOI:** 10.1002/open.202500109

**Published:** 2025-04-28

**Authors:** Svitlana Vitushkina, Ivan Potočňák, Oleksandr Bukrynov, Lucia Váhovská, Mariia Holub, Erik Čižmár

**Affiliations:** ^1^ Department of Materials Physics Institute of Experimental Physics of the Slovak Academy of Sciences Watsonova 47 SK-040 01 Košice Slovakia; ^2^ Department of Inorganic Chemistry P. J. Šafárik University in Košice Faculty of Science Institute of Chemistry Moyzesova 11, SK-041 54 Košice Slovakia; ^3^ Department of Applied Chemistry V. N. Karazin Kharkiv National University Faculty of Chemistry Svobody sq. 4 UA-61022 Kharkiv Ukraine; ^4^ Department of Chemistry Biochemistry and Biophysics University of Veterinary Medicine and Pharmacy in Košice Komenského 73 SK-041 84 Košice Slovakia; ^5^ Synchrotron SOLEIL, L'Orme des Merisiers Départementale 128 FR-91190 Saint-Aubin France; ^6^ P. J. Šafárik University in Košice Faculty of Science Institute of Physics Park Angelinum 9 SK-041 54 Košice Slovakia

**Keywords:** Azides, Copper, Magnetic properties, Structure elucidation, 1,2,4-Triazoles

## Abstract

Two new Cu(II) complexes with abpt (4‐amino‐3,5‐di‐2‐pyridyl‐4*H*‐1,2,4‐triazole) and azido ligands, [Cu(abpt)_2_(N_3_)]NO_3_ (**1**) and [Cu(abpt)_2_(N_3_)_2_]⋅2H_2_O (**2**), have been prepared and characterized by crystal structure analysis, spectral and magnetic measurements. The presence of neutral abpt, as well as azido ligands was proved by IR spectroscopy and the composition of the complexes confirmed an elemental analysis. Monocrystal X‐ray structure analysis revealed that **1** is an ionic pentacoordinated Cu(II) complex, exhibiting a distorted tetragonal pyramidal geometry of the coordination polyhedron, while **2** is a neutral molecular complex with a distorted octahedral environment of the Cu(II) atom. The structures are stabilized by *π*–*π* stacking interactions between the aromatic rings of abpt, as well as various intra‐ and intermolecular hydrogen bonds involving nitrate ions and molecules of solvated water in **1** and **2**, respectively. A field‐induced slow magnetic relaxation was observed at low temperatures in **1**, described by the direct and Raman process involving low‐energy intramolecular vibrational modes, which were predicted by the DFT calculations.

## Graphical abstract

A field‐induced slow magnetic relaxation was observed in an ionic pentacoordinated Cu(II) complex with 4‐amino‐3,5‐di‐2‐pyridyl‐4*H*‐1,2,4‐triazole and azido ligands. The analysis of the relaxation behaviour supports the origin of the slow magnetic relaxation in intramolecular low‐energy vibration modes participating in the one‐phonon direct and two‐phonon Raman process. The field dependence of the low‐frequency relaxation channel suggests the presence of the spatial PB effect.

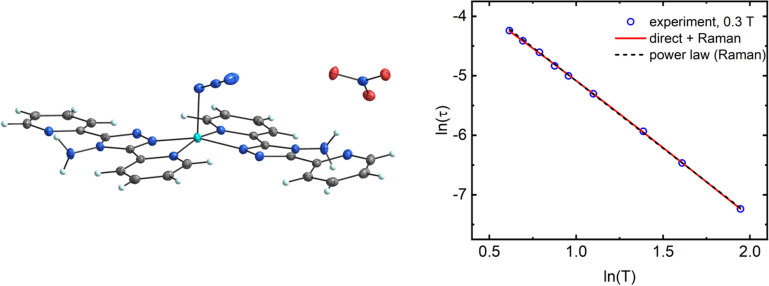

## Introduction

1,2,4‐Triazoles, including 4‐amino‐3,5‐di‐2‐pyridyl‐4*H*‐1,2,4‐triazole (abpt), are versatile ligands in coordination chemistry, particularly known for forming stable complexes with transition metals. The triazole ring combines the coordination properties of pyrazoles and imidazoles and provides multiple coordination sites with the pyridine groups, making abpt a multitopic ligand. This property allows abpt to form diverse and robust metal‐ligand frameworks essential in various applications. In catalysis, these complexes have been used in hydrogenation and cross‐coupling reactions.^[1]^ Abpt derivatives are found in drugs used to treat breast cancer, such as vorozole, letrozole, and anastrozole.^[2]^ The ability of abpt to act as a bridging ligand between two metal centres further enhances its utility in coordination chemistry, particularly in the synthesis of polynuclear complexes.^[3]^ Furthermore, the coordination chemistry of abpt with transition metals has garnered interest for its potential use in spin‐crossover materials^[4]^ and molecular magnetism, particularly in single‐molecule magnets (SMMs).^[5]^

Up to now, our obtained hexacoordinate compounds have been behaving as field‐induced single‐ion magnets with slow relaxation of magnetization, which included different relaxation processes. Thus, pseudooctahedral mononuclear cobalt(II) complex [Co(abpt)_2_(tcm)_2_] (tcm=tricyanmethanide anion) has positive axial and large rhombic anisotropy.^[5a]^ The analysis of the magnetic properties of [Co(abpt)_2_(N_3_)_2_]^[3c]^ revealed negative zero‐field splitting parameter *D*=−24.1 cm^−1^. The X‐band electron paramagnetic resonance gives evidence for a strong rhombic character of the anisotropy. The *ac* susceptibility measurements identified direct, Raman and Orbach relaxation processes. Complexes with dicyanamide ligand (dca), with the formula [M(abpt)_2_(dca)_2_], where M=Cu and Co, also exhibit field‐induced slow spin‐phonon relaxation.^[5c]^ In both complexes, the slow spin‐phonon relaxation is influenced by a severe phonon‐bottleneck (PB) effect that affects the direct process, a dominant relaxation mechanism at low temperatures. The PB effect in [Cu(abpt)_2_(dca)_2_] was suppressed by simply reducing the crystallite size, and analysis of the field dependence of the relaxation time yielded the characteristic energy of vibrational modes of 11 cm^−1^ participating in the Raman process at low magnetic fields. The analysis of magnetic properties and *ab initio* calculations confirmed that [Co(abpt)_2_(dca)_2_] represents a system with a moderate uniaxial anisotropy, yielding an average energy barrier of 82 cm^−1^. The slow magnetic relaxation in the abovementioned complexes appears to be strongly influenced by low‐energy vibrational modes related to the intramolecular molecular rotations and torsion.^[6]^ Their identification is also important for SMMs with high blocking temperature and energy barrier.

Considering the interest in abpt complexes as well as the results of our research of mixed‐ligand complexes of Co(II) and Cu(II) based on abpt and linear or nonlinear pseudohalides, we have focused on the synthesis and magneto‐structural characterization of two novel copper(II) complexes: [Cu(abpt)_2_(N_3_)]NO_3_ (**1**) and [Cu(abpt)_2_(N_3_)_2_]⋅2H_2_O (**2**).

## Results and Discussion

**Scheme 1:**
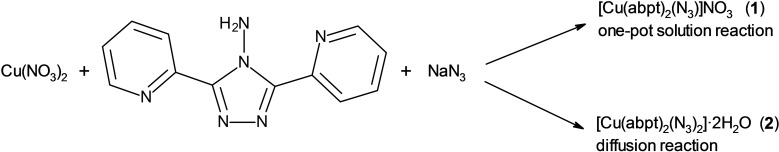
Graphical representation of synthesis of **1** and **2**.

Using Cu(NO_3_)_2_⋅3H_2_O, NaN_3_ and abpt in an one‐pot solution reaction and in a diffusion reaction lead to the formation of two different complexes: [Cu(abpt)_2_(N_3_)]NO_3_ (**1**) and [Cu(abpt)_2_(N_3_)_2_]⋅2H_2_O (**2**), respectively, Scheme 1.

In the IR spectra of **1** and **2**, intense absorptions around 2030 cm^−1^ associated with the asymmetric stretching vibration of the azido ligand ν_*as*_(N₃^−^) were observed. It is known that complex compounds in which the azido ligands are in the *trans*‐ and *fac*‐forms exhibit a single band ν_*as*_(N₃^−^), whereas for less symmetrical *cis*‐ and *mer*‐isomers, this band splits.^[3c]^ In the IR spectrum of **2**, a single band of ν_*as*_(N₃^−^) at 2034 cm^−1^ is observed, indicating *trans*‐positioned azido ligands. As expected, in the spectrum of **1**, which comprises only one azido ligand, a single intense absorption band at 2033 cm^−1^ is observed. These intense absorption bands for **1** and **2** are shifted to the lower wave numbers in comparison with KN_3_ ^[7]^ in consonance with the coordination of the azido ligand, which is in good agreement with similar complexes containing coordinated terminal N₃^−^ ligands.[^3^, ^8^] The bands of medium intensity at 1336 and 1332 cm^−1^ for **1** and **2**, respectively, correspond to the symmetric stretching vibration of ν_*s*_(N₃^−^) and the deformation mode δ(N₃^−^) is observed at 701 cm^−1^ for **1** and 696 cm^−1^ for **2** as medium bands, too.

The presence of conjugated abpt molecules in **1** and **2** was confirmed by characteristic absorption bands of weak intensity: in the region of 3280–3056 cm^−1^ corresponding to the stretching ν(N−H) vibrations of the amino group and in the region of 3197–3077 cm^−1^ corresponding to the stretching ν_*ar*_(C−H) vibrations of aromatic rings. The stretching ν(C=N), ν(C−C), and ν(C−N) vibrations were found in the 1633–1432 cm^−1^ (**1**) and 1639–1430 cm^−1^ (**2**) regions. The out‐of‐plane and in‐plane pyridine ring deformations, which are characteristic for heterocyclic ligands, were located at 420 and 612 cm^−1^, respectively, for **1** and 419 and 611 cm^−1^, respectively, for **2**. These vibrations are found to be positively shifted, suggesting the coordination of the abpt ligand in both prepared compounds.[^3^, ^9^]

In the IR spectrum of **1**, the absorption bands of medium intensity at 1296 and 753 cm^−1^ can be assigned to the stretching ν(NO) and deformation δ(ONO) vibrations of the nitrate anion, respectively.^[10]^ The presence of crystallization water in **2** was confirmed by the broad band of weak intensity of ν(O−H) vibrations at 3439 cm^−1^, by the deformation vibrations δ(HOH) at 1639 cm^−1^ and by the deformation rocking vibration δ_*ρ*_(HOH) at 802 cm^−1^.

The indications obtained from the IR spectra of **1** and **2** were confirmed by the single‐crystal X‐ray structure analysis which has revealed that **1** is an ionic complex formed by [Cu(abpt)_2_(N_3_)]^+^ complex cation and the nitrate counter ion, while **2** is a neutral molecular complex with two molecules of solvated water, [Cu(abpt)_2_(N_3_)_2_]⋅2H_2_O.

### Description of the Structures of [Cu(abpt)_2_(N_3_)]NO_3_ (1) and [Cu(abpt)_2_(N_3_)_2_]⋅2H_2_O (2)

**Figure 1:**
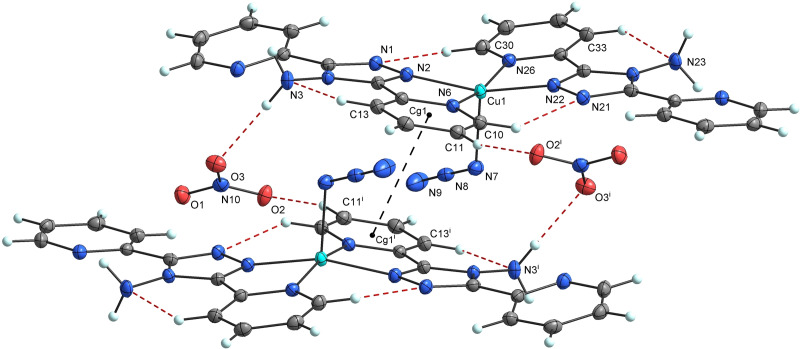
Dimers in crystal structure of **1** with displacement ellipsoids (50 % probability) with selected inter‐ and intramolecular hydrogen bonds (red dashed lines) and *π*–*π* interactions (black dashed lines). Symmetry code: (i)=1 – *x*, 1 – y, 1 – z.

The copper atom in **1** is pentacoordinated and the shape of the coordination polyhedron around it is a distorted tetragonal pyramid as evidenced by the parameter τ=0.09^[11]^ as well as by the results of the SHAPE program, SPY‐5=0.857,^[12]^ Figure 1. The basal plane is formed by the nitrogen atoms of the pyridine (N6 and N26) and triazole (N2 and N22) rings from the two bidentate abpt ligands, while the apical position is occupied by N7 atom of the azido ligand at the distance of 2.165(2) Å. Cu1 atom is displaced from the basal plane, the angles N22–Cu1–N2 and N26–Cu1–N6 are equal to 161.45(6) and 167.07(5)°, respectively (Table 1).

**Table 1:** Selected bond distances and angles (Å, °) for **1** and **2**.

Complex	Bond distances		Bond angles	
**1**	Cu1–N7	2.1653(15)	N22–Cu1–N2	161.45(6)
	Cu1–N22	1.9887(13)	N22–Cu1–N26	80.21(5)
	Cu1–N2	2.0001(14)	N26–Cu1–N6	167.07(5)
	Cu1–N26	2.0454(14)	N2–Cu1–N6	80.10(6)
	Cu1–N6	2.0473(14)	N22–Cu1–N6	96.95(5)
	N7–N8	1.190(2)	N2–Cu1–N26	98.55(5)
	N8–N9	1.162(2)	N22–Cu1–N7	103.07(6)
			N2–Cu1–N7	95.47(6)
			N26–Cu1–N7	93.91(6)
			N6–Cu1–N7	99.02(6)
			N7–N8–N9	178.60(19)
			N8–N7–Cu1	116.97(11)
**2**	Cu1–N6	2.0492(18)	N2–Cu1–N6	80.01(7)
	Cu1–N2	1.9737(19)	N2–Cu1–N2^i^	180.0
	Cu1–N7	2.521(2)	N2–Cu1–N6^i^	99.99(7)
	N7–N8	1.164(3)	N2^i^–Cu1–N7	88.89(8)
	N8–N9	1.186(3)	N2–Cu1–N7	91.11(8)
			N6^i^–Cu1–N7	86.60(7)
			N6–Cu1–N7	93.40(7)
			N7–N8–N9	178.6(3)
			N8–N7–Cu1	115.60(17)

[Symmetry code: (i)=1 – *x*, 2 – *y*, 1 – *z* (**2**)]

The Cu1–N bond lengths involving the pyridyl groups [Cu1–N6=2.047(1) and Cu1–N26=2.045(1) Å] are longer than those corresponding to the triazole rings [Cu1–N2=2.000(1) and Cu1–N22=1.989(1) Å], which is in good agreement with other compounds containing abpt ligand.[^3^, ^5^, ^13^]

The coordinated abpt ligands are not fully planar as evidenced by the dihedral angles between individual rings in these molecules. The dihedral angles between the coordinated pyridyl groups and the triazole rings are 5.2 and 7.9° (for the abpt molecules containing N2/N6, and N22/N26 donor atoms, respectively), while those between the uncoordinated pyridyl groups and the triazole rings are 6.5 and 15.0°, respectively, and those between the two pyridyl groups are 11.6 and 19.8°, respectively. Thus, the second abpt molecule is more twisted. The azido ligand, which is nearly linear [N7–N8–N9=178.60(19)°], is coordinated with a N8–N7–Cu1 angle of 116.97(11)°.

The unequal azide bonds in this complex, N7–N8=1.190(2) and N8–N9=1.162(2) Å, are like those found in [Co(tetraen)N_3_]_2_^+^ (tetraen=tetraethylenepentamine), being 1.209(7) and 1.152(7) Å,^[14]^ [Co{(naph)_2_dien}(N_3_)] ((naph)_2_dien=bis(2‐hydroxy‐1‐naphthaldimine)‐N‐diethylenetriaminedianion), being 1.195(2) and 1.154(2) Å,^[15]^ and in [Co(abpt)_2_(N_3_)_2_] complexes being 1.187(3) and 1.162(3) Å.^[3c]^ In most cases, the longer bond is observed between the donor and the middle nitrogen atoms.

The crystal structure of **1** is stabilized by hydrogen bonds of different types and by *π*–*π* interactions. The abpt molecules exhibit intramolecular C−H⋅⋅⋅N hydrogen bonding between the coordinated pyridyl groups (C10/C30) and the triazole rings (N21/N1), as well as between the coordinated pyridyl groups (C13/C33) and the amino groups (N3/N23), thereby stabilizing the base of the distorted tetragonal pyramid, Figure 1, Table 2. Intermolecular N3–H2N⋅⋅⋅O3 and C11–H11⋅⋅⋅O2^i^ (i=1 – *x*, 1 – *y*, 1 – *z*) hydrogen bonds between abpt molecule and nitrate ions link the neighbouring cations and pack them to form face‐to‐face dimers.

**Table 2:** Inter‐ and intramolecular hydrogen bond interactions in **1** and **2** (Å, °).

Complex	Donor–H⋅⋅⋅Acceptor	D–H	H⋅⋅⋅A	D⋅⋅⋅A	< D−H⋅⋅⋅A
1	C10–H10⋅⋅⋅N21	0.95	2.29	3.081(2)	139.7
	C30–H30⋅⋅⋅N1	0.95	2.38	3.182(2)	141.6
	C13–H13⋅⋅⋅N3	0.95	2.48	3.086(2)	121.7
	C33–H33⋅⋅⋅N23	0.95	2.47	3.085(2)	122.0
	N3–H2N⋅⋅⋅O3	0.89	2.05	2.934(2)	172(2)
	C11–H11⋅⋅⋅O2^i^	0.95	2.30	3.226(2)	165.5
	N23–H3N⋅⋅⋅O3^ii^	0.90(3)	2.20(3)	3.082(2)	169(2)
	N23–H4N⋅⋅⋅O2^iii^	0.89(2)	2.19(2)	3.041(2)	159(2)
	N3–H1N⋅⋅⋅N9^iv^	0.85(3)	2.42(2)	3.057(2)	132(2)
	C38–H38⋅⋅⋅N7^v^	0.95	2.37	3.252(2)	154.6
2	C10–H10⋅⋅⋅N1^i^	0.95	2.33	3.143(3)	143.5
	N3–H2N3⋅⋅⋅N5	0.98(3)	2.14(3)	2.883(3)	131(3)
	C13–H13⋅⋅⋅N3	0.95	2.54	3.142(3)	121.4
	O1–H1O1⋅⋅⋅N9	0.92	1.97	2.879(3)	168.8
	O1–H2O1⋅⋅⋅N9^ii^	0.83	2.08	2.905(3)	175.3
	N3–H1N3⋅⋅⋅O1^iii^	0.93(4)	1.99(4)	2.869(3)	156(3)

[Symmetry codes: (i)=1 – *x*, 1 – *y*, 1 – *z*, (ii)=1 + *x*, 1 + *y*, *z*, (iii)=*x*, 1 + *y*, *z*, (iv)=–1 + *x*, *y*, *z*, (v)=2 – *x*, 2 – *y*, 1 – *z* (**1**); (i)=1 – *x*, 2 – *y*, 1 – *z*, (ii)=1 – *x*, 2 – *y*, 2 – *z*, (iii)=*x*, *y*, –1 + *z* (**2**)]

**Figure 2:**
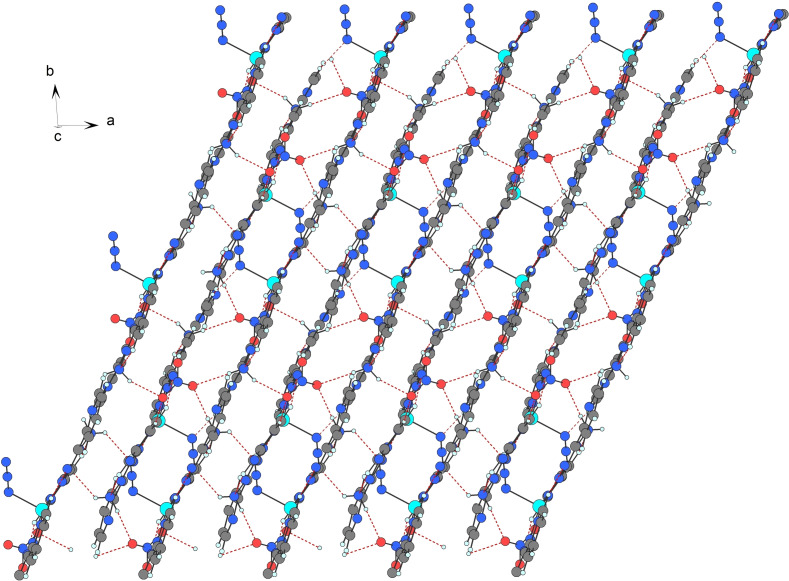
Part of the layer in **1** formed by intermolecular hydrogen bonds (red dashed lines).

Remaining intermolecular hydrogen bonds (Table 2) connect the dimers into infinite layers parallel with the *ab* plane (Figure 2). Further, these layers are stabilized by *π*–*π* interactions (Table 3) between coordinated pyridyl rings of the abpt ligands from neighbouring layers with the centroid‐centroid distance of 3.5032(1) Å as well as between coordinated and uncoordinated pyridyl rings with the centroid‐centroid distance of 3.6048(1) Å to form a 3*D* structure (Figure SI1).

**Table 3:** Cg⋅⋅⋅Cg distances and angles (Å, °) characterizing *π*–*π* interactions in **1** and **2**.

Complex	Cg(I)⋅⋅⋅Cg(J)	Cg⋅⋅⋅Cg	α^a^	β	γ
1	Cg1⋅⋅⋅Cg1^i^	3.5032(1)	0	17.0	17.0
	Cg2⋅⋅⋅Cg3^ii^	3.6048(1)	3	22.8	19.6
2	Cg1⋅⋅⋅Cg2^i^	3.6624(1)	3.9	25.1	24.1

[Symmetry codes: (i)=1 – *x*, 1 – *y*, 1 – *z*; (ii)=1 – *x*, 1 – *y*, – *z* (**1**); (i)=1 + *x*, *y*, *z*; (**2**)] Cg1 represent centroids of the coordinated pyridyl rings containing N6 (**1**) and N5 atoms (**2**); Cg2 represent centroids of the coordinated pyridyl rings containing N26 (**1**) and N6 atoms (**2**); Cg3 represents centroid of the uncoordinated pyridyl ring containing N5 atom (**1**); α^a^ is the dihedral angle between planes I and J. β is the angle between Cg(I)⋅⋅⋅Cg(J) vector and normal to plane I. γ is the angle between Cg(I)⋅⋅⋅Cg(J) vector and normal to plane J.

**Figure 3:**
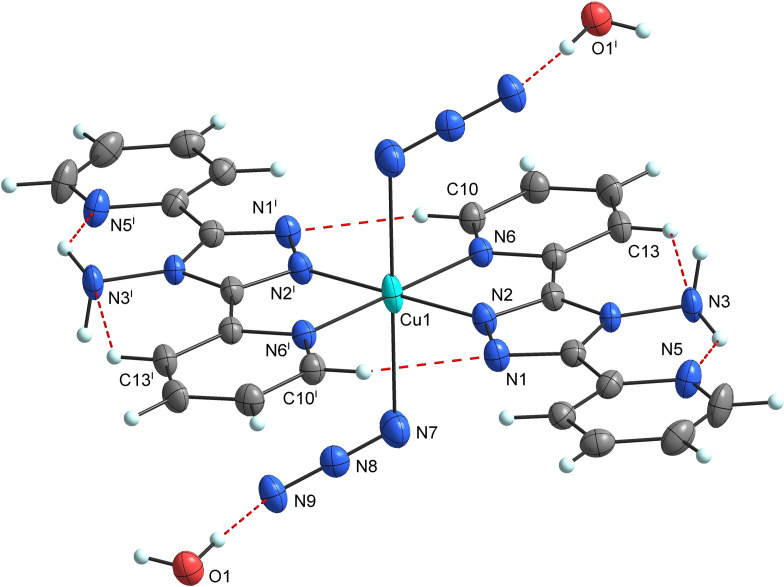
Molecular structure of **2** with displacement ellipsoids (50 % probability) with selected inter‐ and intramolecular hydrogen bonds (red dashed lines). Symmetry code: (i)=1 – *x*, 2 – *y*, 1 – *z*.

The asymmetric unit of **2** contains one molecule of abpt and one azido ligand coordinated to the copper atom, as well as one molecule of solvated water (Figure 3). Due to the centre of inversion located at the central atom, the coordination number of Cu1 atom is 6 and the central atom is in a distorted octahedral {CuN6} environment, of which the four basal nitrogen atoms coordinated to Cu1 atom belong to the pyridyl (N6 and N6^i^) and triazole (N2 and N2^i^) (i=1 – *x*, 2 – *y*, 1 – *z*) groups of the two bidentate abpt ligands. The axial positions are occupied by two terminally *trans*‐coordinated nitrogen atoms from the azido ligands with the Cu1–N7 distance of 2.521(2) Å, which is much longer due to the Jahn–Teller effect than the Cu1–N bond distances in the basal plane (Table 1). The Cu1–N bond lengths involving the pyridyl groups [Cu1–N6=2.049(2) Å] are longer than those corresponding to the triazole rings [Cu1–N2=1.974(2) Å], which is in good agreement with other isostructural compounds containing abpt ligand.[^3^, ^5^, ^13^] Coordinated abpt molecules in **2** are approximately planar. All dihedral angles in **2** are smaller compared to the corresponding ones in the abpt ligands in **1**. The dihedral angles between the coordinated and uncoordinated pyridyl groups and the triazole ring are cca. 3.1–3.9°. The angular distortions of coordination octahedron are determined by the chelate binding of abpt ligands and result in the expected substantial deviations of the chelate bite angle N2–Cu1–N6=80.01(7)° from 90° (Table 1). The octahedral distortion parameter Σ=(|90 – ϕ_i_|), where ϕ_i_ represents the twelve *cis* N−Cu1–N angles, is equal to 58.02°. The distortion of the coordination polyhedron is also confirmed by the results of the SHAPE program, OC‐6=1.739.^[12]^ The nearly linear azido ligand [N7–N8–N9=178.6(3)°] is coordinated with a N8–N7–Cu1 angle of 115.60(17)°, these angles are similar to those found in **1**. The unequal azide bonds in **2**, N7–N8=1.164(3) Å and N8–N9=1.186(3) Å, have the opposite sequence of the bond lengths than in **1**.

**Figure 4:**
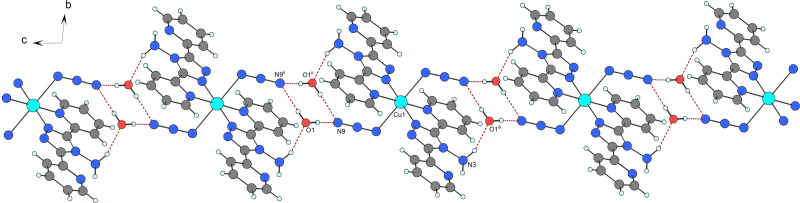
Part of the chain in **2** formed by intermolecular hydrogen bonds (red dashed lines). Intramolecular hydrogen bonds are omitted for clarity. Symmetry code: (ii)=1 – *x*, 2 – *y*, 2 – *z*, (iii)=*x*, *y*, –1 + *z*.

The planar coordination of the abpt molecules in **2** is stabilized by intramolecular hydrogen bonds (Figure 3, Table 2). Weak C−H⋅⋅⋅N hydrogen bonds are formed between the coordinated pyridine groups (C10/C10^i^, i=1 – *x*, 2 – *y*, 1 – *z*) and the triazole rings (N1/N1^i^), while strong N−H⋅⋅⋅N bonds are formed between the amine H atoms (H2/H2^i^ the donor atoms N3/N3^i^) and the uncoordinated pyridine groups (N5/N5^i^) of abpt. Besides, the coordinated pyridyl groups (C13/C13^i^) are hydrogen bonded to the amine groups (N3/N3^i^) of the abpt molecules by C−H⋅⋅⋅N hydrogen bonds, similar to **1**. Water molecules actively participate in the formation of the strong intermolecular hydrogen bonds with the azido ligands (O1–H1O1⋅⋅⋅N9 and O1–H2O1⋅⋅⋅N9^ii^, ii=1 – *x*, 2 – *y*, 2 – *z*) and with the amine H atom (N3–H1N3⋅⋅⋅O1^iii^, iii=*x*, *y*, –1 + *z*) of the abpt molecules (Table 2). Intermolecular hydrogen bonds involving water molecules lead to the formation of a chain along the *c* axis (Figure 4). In the crystal structure of **2**, a weak *π*–*π* interaction is observed between the coordinated and uncoordinated pyridine rings of the abpt molecules from the neighbouring chains with the centroid‐centroid distance of 3.6624(1) Å (Table 3). Due to these interactions, the chains formed by hydrogen bonds are linked together to create a layer parallel with the *ac* plane (Figure SI2).

### Magnetic Properties of 1

**Figure 5:**
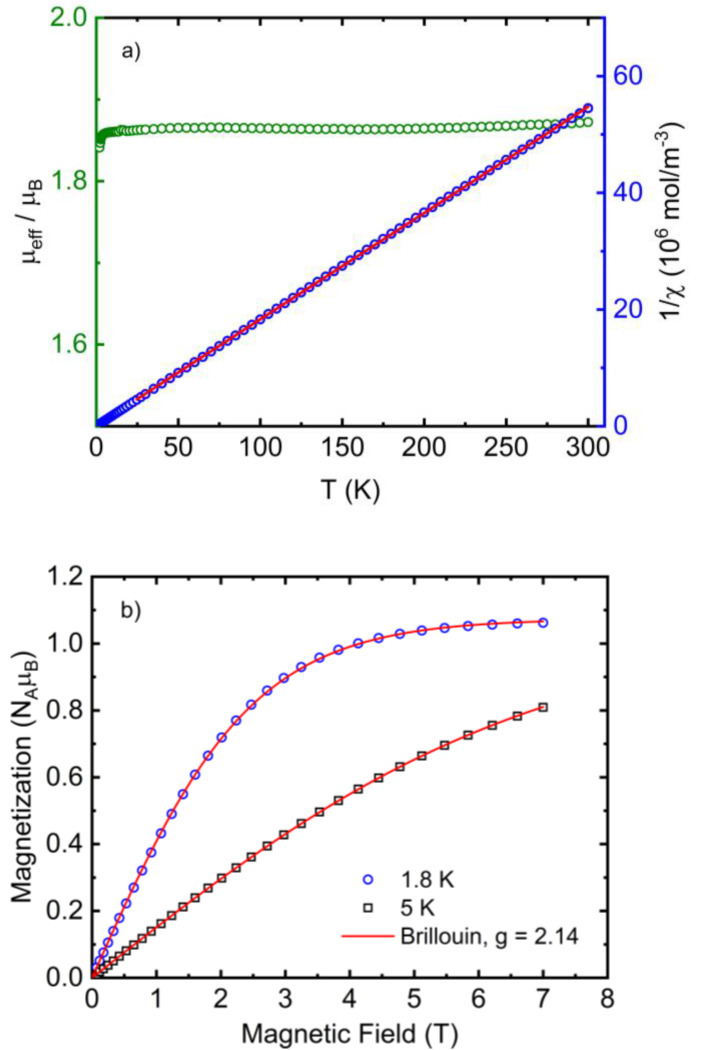
a) The temperature dependence of the effective magnetic moment (green symbols) and 1/*χ* (blue symbols) of **1** and the Curie‐Weiss fit to 1/*χ* (red solid line). b) Field dependence of magnetization of **1** measured at 1.8 K (blue circles) and 5 K (black squares), including the Brillouin function for the ideal paramagnet (red solid lines).

The temperature dependence of the effective magnetic moment *μ*_eff_ (or *χT*) of **1** is characterized by a slight decrease at very low temperatures below ≈ 8 K (Figures 5a and SI3a). Such behaviour suggests the presence of weak antiferromagnetic (AFM) exchange interactions. The room temperature value of *μ*_eff_=1.87 *μ*_B_ is typical for Cu(II) ions (*μ*_B_ is Bohr magneton). A fit of the Curie‐Weiss law to the inverse susceptibility in the temperature range 25–300 K yields a small value of the Weiss temperature of Θ=‐0.6 K with the field dependence of the magnetization measured at 1.8 K and 5 K well described by the Brillouin function for the paramagnet with an average *g*‐factor *g*=2.14 (Figure 5b). Such behaviour shows that bulky abpt ligands prevent significant exchange interaction between Cu(II), which is similar to another complex with abpt ligand [Cu(abpt)_2_(dca)_2_] that exhibit field‐induced slow spin‐phonon relaxation.^[5c]^ Such a weak exchange interaction is due to the spatial orientation of the magnetic orbital $d_{x^{2}-y^{2}}$ in the equatorial plane created by abpt ligands in the coordination square‐pyramidal environment. The evidence of the elongated square‐pyramidal coordination due to the Jahn‐Teller effect was obtained from the EPR spectra of **1** measured in the X‐band EPR spectrometer at 2 K (Figure SI3b). The EPR spectra were analysed using EasySpin Toolbox,^[16]^ yielding the set of *g*‐factors [*g*_x_, *g*_y_, *g*_z_]=[2.056, 2.066, 2.265], with average value *g*=2.135. An anisotropic line broadening [*ΔB*_x_, *ΔB*_y_, *ΔB*_z_]=[530, 118, 1182] MHz was introduced in the simulation to include the effect of the unresolved hyperfine splitting. The obtained results, the smallest *g*‐factor component *g_x_* >2.04 and *g_x_*, *g_y_* < *g_z_* are compatible with a tetragonal deformation of crystal field at the Cu(II) site in the square‐pyramidal coordination.^[17]^

To support the estimation of the weak exchange interactions mediated through hydrogen bonds and *π*–*π* interactions in **1** we performed simple broken symmetry DFT calculations using a pair of molecules with NO_3_ anions as shown in Figure 1. We used B3LYP and M06 exchange‐correlation functionals obtaining weak AFM exchange interaction *J/k_B_* of −0.32 K and −0.48 K, respectively, with most of the spin density localized in Cu(II) ions and nitrogens in the equatorial plane. This is in good agreement with the molecular‐field estimate of effective exchange interaction from Curie‐Weiss fit as *zJ*=4Θ, where *z* is the number of the nearest neighbors, if we assume four nearest neighbors for each spin in the layers formed by hydrogen bonds and *π–π* interactions.

**Figure 6:**
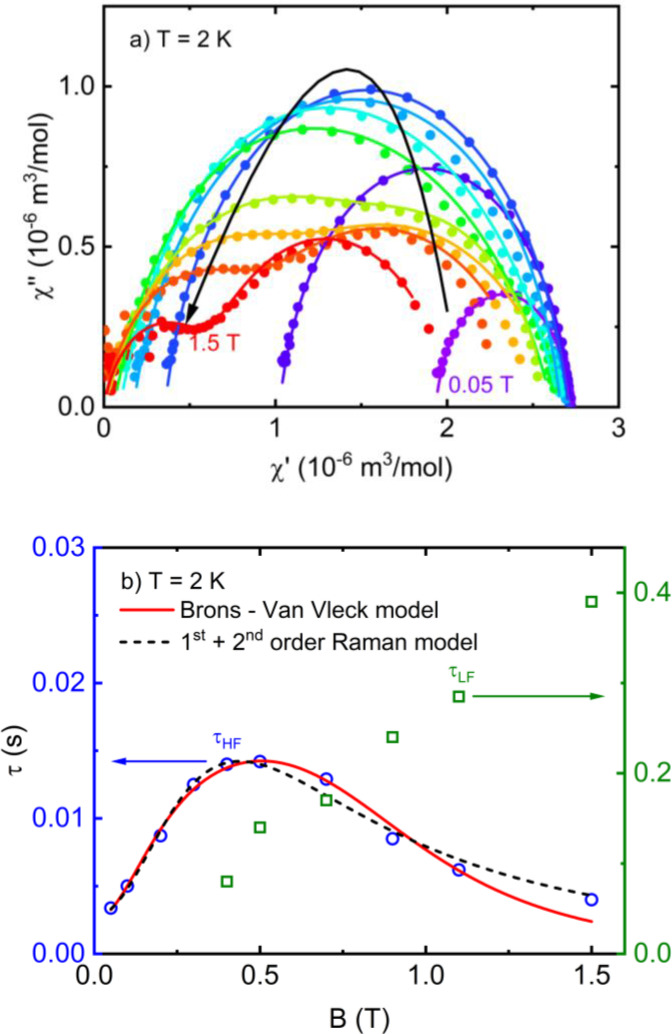
a) Field‐dependent Cole‐Cole plots of **1** obtained at 2 K (symbols), including the fits using the modified Debye model (solid lines of corresponding colour) for selected magnetic fields. b) Field‐dependence of the relaxation time of two relaxation channels extracted using a modified Debye model Equation SI1 (open symbols). The field dependence of *τ*_HF_ (blue circles) was analysed using the Brons‐Van Vleck model, Equation 1 (red solid line) and model including 1^st^ and 2^nd^‐order Raman process, Equation 2 (black dashed line).

Motivated by the observation of slow magnetic relaxation in Cu(II)‐based complexes with the unusual reciprocating temperature dependence of the relaxation time^[18]^ or a complex influence of the PB effect in slow magnetic relaxation of Kramers ions^[5c]^ we have also performed *ac* susceptibility studies in **1**. The *ac* susceptibility measurements were performed at various applied *dc* magnetic fields to search for slow magnetic relaxation at 2 K. Only after the application of non‐zero *dc* magnetic field a slow relaxation of magnetization in the frequency range from 0.1 Hz to 1 kHz appeared as a maximum in the frequency dependence of the out‐of‐phase component of *ac* susceptibility, *χ*′′, see Figure SI4. At *dc* magnetic fields above 0.5 T, another low‐frequency relaxation channel appears (or splits from the original low‐field channel). The Cole‐Cole plots were then obtained in the magnetic fields up to 1.5 T (Figure 6a), and the field dependence of the relaxation time shown in Figure 6b was extracted using a modified Debye model Equation SI1.^[19]^

The relaxation time of the low‐frequency (LF) relaxation channel *τ*_LF_ gradually increases up to high magnetic fields, which seems to be a result of the so‐called spatial PB effect (associated with the combined effect of a large magnetic specific heat and poor thermal contact) influencing the direct relaxation process, as shown for other mononuclear complexes showing slow magnetic relaxation.[^6^, ^20^] In our previous work^[5c]^ on [Cu(abpt)_2_(dca)_2_] complex, this effect could be suppressed by reducing the size of the crystallites to study intrinsic relaxation mechanisms. Decreasing the crystallite size yields a larger surface that allows better thermal contact with the exchange gas, and the spatial PB effect can be suppressed.^[6a]^ In such a comprehensive study, we could suppress the PB effect, extract the typical frequency of the phonons participating in the Raman process, and relate it to the characteristic vibrational modes of the molecular groups. For complex **1**, the experiments were performed using a fine polycrystalline sample; we can speculate that the part of the sample is still affected by the spatial PB effect evidenced by the formation of LF relaxation with such field dependence of the relaxation time; or the direct process is separated in distinct relaxation channel, and the high‐frequency (HF) relaxation channel contains the Raman process only.

The HF relaxation channel in changing *dc* magnetic field behaves according to the Brons‐Van Vleck model (see also Equation SI2:[^6^, ^20^]

(1) $$\tau^{-1}=cB^{4}+d\frac{1+eB^{2}}{1+fB^{2}}$$

proposed to describe the combination of the direct and field‐dependent Raman processes in Kramers systems with no energy barrier. The first term accounts for the field dependence of the direct mechanism of relaxation between two states of Kramers doublet split by the Zeeman energy, which is included in *c*=*AT*. Intermolecular interactions in the structure (weak exchange interactions mediated by hydrogen bonds in the structure of **1** or dipolar interactions) or intramolecular interactions (spin‐nuclei/ hyperfine interactions) may create internal fluctuating fields mixing the levels of Kramers doublet, which is included in the second term. Spin‐nuclei/hyperfine interaction breaks the time‐reversal symmetry of Kramers doublet and lifts van Vleck's cancelation rule allowing a direct relaxation process at low temperatures if low energy phonons are present. The prefactor *d*=*CT* ^*n*^ defines the zero‐field Raman process. While the direct process is more efficient at higher fields where phonons with higher frequency can participate in spin‐phonon relaxation, the Raman process will dominate at low *dc* fields. The fit of Equation 1 to the field dependence of the relaxation time of the HF channel *τ*_HF_ in Figure 6b yielded *A*=35.7 K^−1^s^−1^, *d*=390.3 s^−1^, *e*=19.2 T^−2^, and *f*=133.6 T^−2^. On the other hand, such a field dependence of the relaxation time can be described by another model proposed for anisotropic SMMs with large relaxation barriers and large blocking temperatures,^[21]^ which shows that the 1^st^ and 2^nd^‐order Raman process governs low‐temperature relaxation. While the 1^st^‐order Raman process is field independent, the 2^nd^‐order Raman process through the virtual excited state is governed by quadratic field dependence. The model includes the Brons‐Van Vleck prefactor to include the influence of internal fluctuating fields in the form

(2) $$\tau^{-1}=\frac{\mu \sigma_{B}^{2}/2+B^{2}}{\sigma_{B}^{2}/2+B^{2}}\left(c_{1}T^{n}+c_{2}T^{m}B^{2}\right),$$

where *μ* and *σ*_B_ depend on the unknown internal fields, *n* and *m* are determined by the phonon density of states and the lowest crystal‐field gap. As shown in Figure 6b, Equation 2 describes well the field dependence of *τ*_HF_ in **1** with *μ*=19.6, *σ*_B_=0.148 T, *c*_1_=2.81 K^−n^s^−1^, *c*_2_=5.99 T^−2^K^−n^s^−1^ with exponents *n*=2.72 and *m*=3.84 often observed in Kramers systems with the involvement of low energy optical phonons or local intermolecular vibrations in the Raman process.[^6^, ^22^] However, the 2^nd^‐order Raman process could not be effective for spin‐1/2 ions as Cu(II) because the relaxation rate is inversely proportional to the excitation energy of the next excited crystal field state. The excited electronic states of Cu(II) ion lie at about tens of thousands of cm^−1^; thus, it lacks excited spin states able to act as virtual states in the relaxation process. In addition, the exchange interactions are too weak to create a collective state with high‐energy excitations in the studied temperature range.

**Figure 7:**
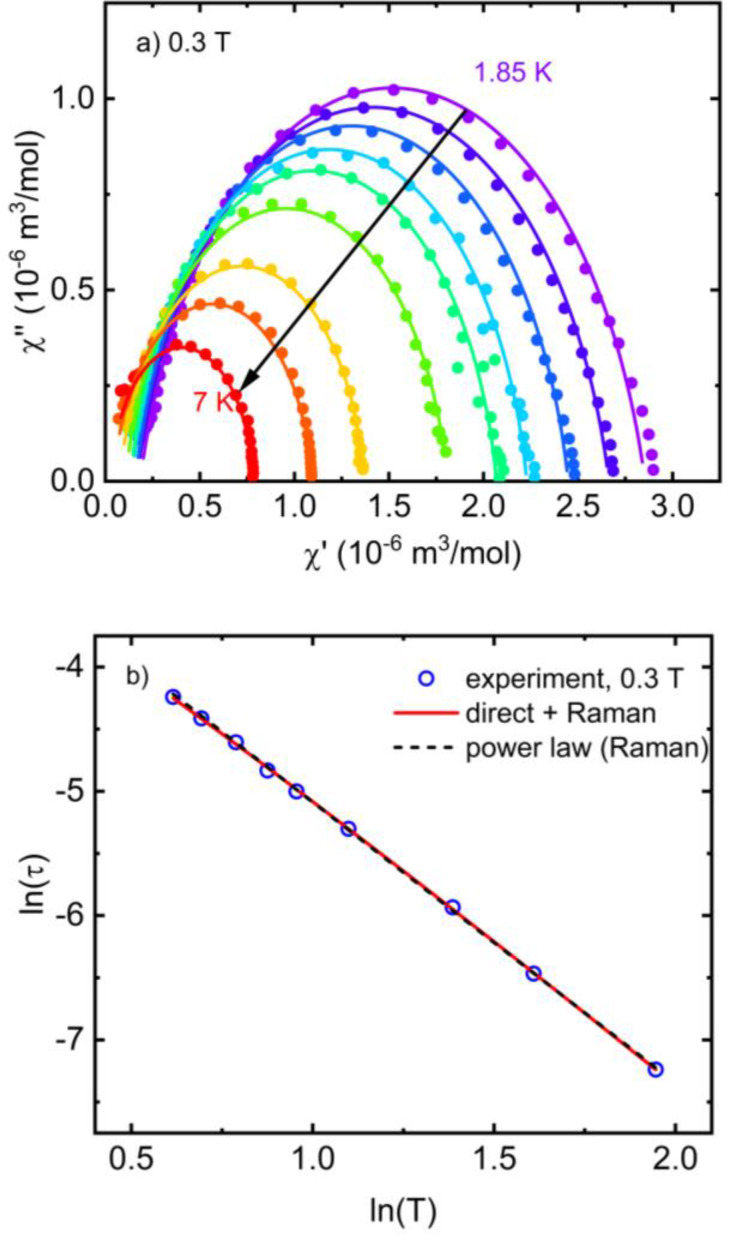
a) Temperature‐dependent Cole‐Cole plots (symbols) of **1** obtained in the *dc* magnetic field of 0.3 T, including the fits (solid lines) using the modified Debye model Equation SI1. b) Temperature dependence of the relaxation time in logarithmic representation (symbols). The red solid line corresponds to Equation 3 (direct and Raman process), and the black dashed line to Equation 4 (power law, Raman).

Following the study of slow relaxation of magnetization in different *dc* magnetic fields, the *dc* field of 0.3 T, when presumably only a single relaxation channel was observed, was chosen to study the temperature dependence of the relaxation time. The relaxation could be traced up to 7 K in the frequency dependence of *χ*′′ (Figure SI5), even with a small signal detectable at 10 K. The corresponding Cole‐Cole plots are shown in Figure 7a, and the temperature dependence of the relaxation time extracted using Equation ESI1 is plotted in Figure 7b. Taking into account the contributions from the direct and Raman relaxation processes, which are theoretically possible in Cu(II) complexes, the temperature dependence of the relaxation time was modelled using the equation

(3) $$\tau^{-1}=AT+CT^{n},$$

where *A* and *C* parameters describe the probability of direct and Raman relaxation processes. If only the Raman process is assumed for the HF relaxation channel, one can use a simplified power law formula4

(4) $$\tau^{-1}=CT^{n}.$$

The fits of Equation 3 and Equation 4 to the experimental data are shown in Figure 7b with *A*=7.14 K^−1^s^−1^, *C*=13.19 K^−n^s^−1^, and *n*=2.38 for the presence of direct and Raman process or with *C*=16.9 K^−n^s^−1^, and *n*=2.26 for the presence of Raman process only. The slow magnetic relaxation with such a low exponent for the Raman process was observed in similar complexes when the relaxation process was explained by the presence of low‐energy vibrational modes with a strong spin‐phonon coupling to affect the slow spin‐phonon relaxation in several vanadyl‐based or other complexes.^[6]^ Such vibrational modes are related to intramolecular molecular rotations and torsion. They can be effectively observed by inelastic X‐ray scattering down to 1–2 meV (∼8‐16 cm^−1^) as shown, e. g., in VO(TPP) molecular qubit complex.^[6d]^ Those findings show how to design new SMMs to avoid direct and Raman relaxation mechanisms using rigid ligands to suppress low‐energy vibrational modes and promote the over‐the‐barrier Orbach relaxation process only. Calculating the exact phonon spectra or spin‐phonon coupling using DFT methods is still a computationally demanding task. Nevertheless, the presence of low‐energy intramolecular vibrational modes participating in the low‐temperature direct and Raman relaxation process can be estimated by DFT calculations on isolated molecules as implemented in ORCA. For such calculations, the geometry of the isolated molecule of **1** and **2** were optimized using TPSS functional, and vibrational modes were calculated. The geometry optimized in the gas phase yields some deviation from the structure in the crystal packing, but it can give some insight into the vibrational spectra. The lowest calculated energies of vibrational modes of 6.6, 16.99, 20.89, 36.96, 38.77, 44.46, and 45.63 cm^−1^ for **1** and 10.41, 19.14, 29.14, 30.14, 37.05, 40.23, and 48.41 cm^−1^ for **2** correspond to vibrations of abpt ligands (only modes with energy less than 50 cm^−1^ are reported). Some of the calculated modes involve the central ion and its first coordination sphere with the probability of sufficient spin‐phonon coupling to participate in the low‐temperature spin‐phonon relaxation. Figure SI6 depicts the modes with the largest displacement vectors in the first coordination sphere of the central ion (16.99 cm^−1^ mode for **1** and 29.14 cm^−1^ mode for **2**) that involve the ligand atoms in the equatorial plane of the coordination polyhedron where the unpaired electron resides in the magnetic $d_{x^{2}-y^{2}}$ orbital.

## Conclusions

Two new copper complexes with abpt and azido ligands, [Cu(abpt)_2_(N_3_)]NO_3_ (**1**) and [Cu(abpt)_2_(N_3_)_2_]⋅2H_2_O (**2**), were synthesized and characterized by infrared spectroscopy, elemental, and single‐crystal X‐ray analysis. The structural analysis showed that **1** is an ionic pentacoordinated complex whose coordination polyhedron is a distorted tetragonal pyramid with nitrogen atoms of abpt ligands occupying the basal plane, while in the apical position the azido ligand is coordinated. On the other hand, **2** is a neutral molecular hexacoordinated complex with a distorted octahedral polyhedron. In **1**, hydrogen bonds between the abpt molecules and nitrate ions link neighbouring cations, arranging them in a face‐to‐face orientation to form dimers, which are linked by intermolecular hydrogen bonds to create infinite layers. These layers are further stabilized by *π*–*π* interactions, resulting in a 3*D* structure. In the crystal structure of **2**, intermolecular bonds involving water molecules stabilize the formation of a chain. Weak *π*–*π* interactions between the aromatic rings of the abpt molecules further link the chains formed by hydrogen bonds what results in the formation of the layered structure. A field‐induced slow magnetic relaxation was observed in **1**, with two relaxation channels emerging at high magnetic fields. The analysis of the field dependence and temperature dependence of the relaxation time agrees with recent predictions identifying the origin of the slow magnetic relaxation in intramolecular low‐energy vibration modes participating in the one‐phonon direct and two‐phonon Raman process. The field dependence of the low‐frequency relaxation channel suggests that the spatial phonon‐bottleneck effect also affects the relaxation of magnetization in **1**.

## Experimental part

### Materials and Physical Techniques

To prepare the studied complexes, Cu(NO_3_)_2_⋅3H_2_O, 99 % from Acros Organics was used. The ligands 4‐amino‐3,5‐di‐2‐pyridyl‐4*H*‐1,2,4‐triazole, 97 % and NaN_3_, 99.5 % were received from Sigma‐Aldrich and Acros Organics, respectively; the solvents (ethanol, 99.8 %, methanol 99.5 % and acetonitrile, 99.8 %) used in the syntheses were received from Centralchem, mikroCHEM and J.T. Baker, respectively. All chemicals were used without further purification. The infrared spectra of the complexes were recorded in the 4000–400 cm^−1^ region, using KBr pellets on Nicolet 6700 FT‐IR spectrophotometer (Thermo Scientific). Elemental analysis of C, H and N was carried out using a CHNOS Elemental Analyzer vario MICRO (Elementar Analysen systeme GmbH).

Static (*dc*) and alternating‐current (*ac*) magnetic measurements were performed in a commercial Quantum Design MPMS®3 magnetometer in temperatures ranging from 1.8 to 300 K in magnetic fields up to 7 T. The *dc* susceptibility was estimated as the ratio of the magnetic moment and applied magnetic field of 0.1 T. The *ac* susceptibility measurements covered the excitation frequency range from 0.1 Hz to 1 kHz. A fine powder specimen was fixed in a gelatine capsule held by a clear straw. The signal contribution of the gelatine capsule and the diamagnetic contribution of the sample estimated using Pascal's constants^[23]^ were subtracted from raw data. The electron paramagnetic resonance (EPR) spectra were studied using Bruker ELEXSYS II E500 X‐band spectrometer with an operating frequency of 9.4 GHz and ESR910 helium flow–type cryostat. Part of the sample from magnetic measurements was mixed with Apiezon N grease and attached to the Suprasil sample holder. Due to a small amount of **2** available, only the magnetic properties of **1** were studied in detail.

## Computational Details

Vibronic properties were calculated using FREQ keyword as implemented in the ORCA 5.0.4 computational package^[24]^ from the optimized geometry of the isolated molecule in the gas phase obtained by the DFT method using TPSS functional^[25]^ with def2‐TZVP^[26]^ and def2/J^[27]^ basis sets, including the atom‐pairwise dispersion correction with the Becke‐Johnson damping scheme (D3BJ).^[28]^ The broken‐symmetry DFT calculation^[29]^ was done using the B3LYP^[30]^ and M06^[31]^ exchange‐correlation functionals. The exchange coupling was obtained from a single‐point approach (using X‐ray determined structure) using the Yamaguchi formalism.^[32]^ The values of exchange interactions are reported using $ℋ=-J{\hat{S}}_{1}{\hat{S}}_{2}$ Hamiltonian. Tight SCF convergence criteria were used.

### Syntheses

#### Synthesis of [Cu(abpt)_2_(N_3_)]NO_3_ (1)

A mixture of abpt (214 mg, 0.9 mmol) and NaN_3_ (59 mg, 0.9 mmol) dissolved in methanol (60 mL) was heated and stirred for about 15 min. To this hot mixture, a solution of Cu(NO_3_)_2_⋅3H_2_O (97 mg, 0.4 mmol) in 80 mL water was added. The resulting green solution was mixed 10 min under heating, filtered and then allowed to crystallize at room temperature. After 10 days, dark green tabular crystals of **1** suitable for X‐ray analysis were collected by filtration, washed with methanol and dried in air. Yield: 208 mg (81 %) based on Cu(NO_3_)_2_⋅3H_2_O.

Elemental analysis (%): Calc. for C_24_H_20_CuN_16_O_3_ (644.11) (**1**) (%): C, 44.76; H, 3.13; N, 34.80; Found: C, 44.58; H, 3.22; N, 34.93. IR (KBr, cm^−1^): 3280(w), 3101(w), 3056(w), 2033(s), 1633(m), 1609(m), 1586(m), 1570(m), 1522(w), 1499(m), 1461(m), 1450(m), 1432(m), 1384(m), 1364(m), 1336(m), 1296(m), 1260(m), 1153(w), 1098(w), 1054(m), 1035(m), 994(w), 802(s), 753(m), 701(m), 647(w), 638(w), 612(w), 420(w), 400(w).

#### Synthesis of [Cu(abpt)_2_(N_3_)_2_]⋅2H_2_O (2)

Single crystals of this compound were obtained at the interface of a layered system, with the lower layer comprising an aqueous solution (50 mL) of Cu(NO_3_)_2_⋅3H_2_O (60 mg 0.25 mmol) and the upper layer comprising abpt (143 mg, 0.6 mmol) and NaN_3_ (39 mg, 0.6 mmol) dissolved in a mixture of acetonitrile (40 mL) and ethanol (20 mL). This layered system was allowed to stand undisturbed in a dark place at room temperature. Dark green needles of **2**, suitable for X‐ray analysis were obtained in the bottom of the test tube after 20 days. The crystals were collected by filtration, washed with ethanol and dried in air. Yield: 25 mg (15 %) based on Cu(NO_3_)_2_⋅3H_2_O.

Elemental analysis (%): Calc. for C_24_H_24_CuN_18_O_2_ (660.16) (**2**) (%): C, 43.67; H, 3.66; N, 38.19; Found: C, 43.85; H, 3.45; N, 38.03. IR (KBr, cm^−1^): 3439(sh), 3197(w), 3077(w), 2034(s), 1639(m), 1611(m), 1589(m), 1570(m), 1522(w), 1499(m), 1464(m), 1430(m), 1384(s), 1332(m), 1304(m), 1260(m), 1153(w), 1102(w), 1057(m), 1018(m), 802(s), 752(m), 727(w), 696(m), 646(w), 639(w), 611(w), 489(w), 461(w), 419(w).

#### Crystallography

Diffraction data for crystal structures were collected using an Agilent SuperNova Dual (**1**) and Oxford Diffraction Xcalibur Gemini ultra (**2**) diffractometers, both equipped with an Atlas S2 CCD detector, using CuKα radiation. CrysAlis PRO 1.171.40.67a^[33]^ was used for data collection, cell refinement, data reduction and absorption corrections. The structures were solved by SHELXT^[34]^ and subsequent Fourier syntheses using SHELXL2018,^[35]^ implemented in WinGX program suit.^[36]^ Anisotropic displacement parameters were refined for all non‐H atoms. All aromatic carbon bonded hydrogen atoms were placed in the calculated positions [C–H=0.95 Å] and refined riding on their parent C atoms, with *U*_iso_(H)=1.2U_eq_(C). Hydrogen atoms of the amine group in **1** and **2** were found in the difference map and then refined using a riding model. An analysis of bond distances and angles was performed using SHELXL2018 while PLATON^[37]^ running under WinGX was used to analyse the *π*–*π* interactions. DIAMOND^[38]^ was used for molecular graphics. The unit cell parameters, crystal and refinement data for **1** and **2** are summarized in Table SI1.

Diffraction analysis for powder sample **1** was done on a Rigaku powder diffractometer (Rigaku Ultima IV X‐ray powder diffractometer with the Rigaku DteX Ultra Silicon micro‐strip detector), utilizing CuKα radiation. A comparison of the measured diffraction pattern of **1** with the diffraction pattern calculated from the crysal structure of **1** is shown in the Figure SI7.

## Conflict of Interests

The authors declare no conflict of interest.

## Supporting information

As a service to our authors and readers, this journal provides supporting information supplied by the authors. Such materials are peer reviewed and may be re‐organized for online delivery, but are not copy‐edited or typeset. Technical support issues arising from supporting information (other than missing files) should be addressed to the authors.

Supporting Information

## Data Availability

The data that support the findings of this study are available in the supplementary material of this article.
