# Supplementary material for: Penta‐ and Hexacoordinated Copper(II) Complexes with Azido and 4‐amino‐3,5‐di‐2‐pyridyl‐4H‐1,2,4‐triazole Ligands with Field‐Induced Slow Magnetic Relaxation
Source: ChemistryOpen. 2025 Apr 28;14(10):e202500109. doi: 10.1002/open.202500109 (PMC12518045; doi:10.1002/open.202500109)
Supplement: Supplementary file 1 — Supporting Information [file OPEN-14-e202500109-s001.pdf]

ChemistryOpen

Supporting Information

Penta- and Hexacoordinated Copper(II) Complexes with Azido and 4-amino-3,5-di-2-pyridyl-4*H*-1,2,4-triazole Ligands with Field-Induced Slow Magnetic Relaxation

Svitlana Vitushkina,* Ivan Potočňák, Oleksandr Bukrynov, Lucia Váhovská, Mariia Holub, and Erik Čižmár*

RESEARCH ARTICLE

Penta- and Hexacoordinated Copper(II) Complexes with Azido and 4-amino-3,5-di-2-pyridyl-4*H*-1,2,4-triazole ligands with Field-induced Slow Magnetic Relaxation

Svitlana Vitushkina,*[a,c] Ivan Potočňák,[b] Oleksandr Bukrynov,[c] Lucia Váhovská,[d] Mariia Holub,[e] Erik Čižmár*[f]

[a] S. Vitushkina
Department of Materials Physics
Institute of Experimental Physics of the Slovak Academy of Sciences
Watsonova 47, SK-040 01 Košice, Slovakia
E-mail: vitushkina@saske.sk

[b] I. Potočňák
Department of Inorganic Chemistry
P. J. Šafárik University in Košice, Faculty of Science, Institute of Chemistry
Moyzesova 11, SK-041 54 Košice, Slovakia

[c] S. Vitushkina, O. Bukrynov
Department of Applied Chemistry
V. N. Karazin Kharkiv National University, Faculty of Chemistry
Svobody sq. 4, UA-61022 Kharkiv, Ukraine

[d] L. Váhovská
Department of Chemistry, Biochemistry and Biophysics
University of Veterinary Medicine and Pharmacy in Košice
Komenského 73, SK-041 84 Košice, Slovakia

[e] M. Holub
Synchrotron SOLEIL, L'Orme des Merisiers
Départementale 128, FR-91190 Saint-Aubin, France

[f] E. Čižmár
P. J. Šafárik University in Košice, Faculty of Science, Institute of Physics
Park Angelinum 9, SK-041 54 Košice, Slovakia
E-mail: erik.cizmar@upjs.sk

WILEY-VCH

Table SI1. Crystal data and structure refinement for **1** and **2**.

	**1**	**2**
Empirical formula	$C_{24}H_{20}CuN_{16}O_3$	$C_{24}H_{24}CuN_{18}O_2$
Formula weight	644.11	660.16
Temperature	95(2) K	120(2) K
Wavelength	1.54184 Å	1.54184 Å
Crystal system	Triclinic	Triclinic
Space group	*P*-1	*P*-1
Unit cell dimensions	*a* = 7.18315(12) Å	*a* = 7.3630(2) Å
	b = 12.78604(20) Å	*b* = 8.4261(3) Å
	c = 14.2367(2) Å	*c* = 11.0044(4) Å
	α = 92.9770(12)°	*α* = 96.046(3)°
	β = 102.3189(13)°	*β* = 93.551(3)°
	γ = 93.8350(13)°	*γ* = 94.397(3)°
Volume	1271.65(4) $Å^3$	675.24(4) $Å^3$
Z	2	1
Density (calculated)	1.682 $Mg.m^{-3}$	1.623 $Mg.m^{-3}$
Absorption coefficient	1.780 mm^{-1}	1.683 mm^{-1}
F(000)	658	339
Crystal size	0.099x0.086x0.029mm^3	0.325x0.205x0.175mm^3
Θ range for data collection	3.472 to 74.464°	4.050 to 67.646°
Index ranges	-8≤*h*≤8, -15≤*k*≤15, -7≤*l*≤17	-8≤*h*≤8, -10≤*k*≤10, -13≤*l*≤13
Reflections collected	20226	10442
Independent reflections	5106 [*R*(int) = 0.0224]	2421 [*R*(int) = 0.0293]
Data / restraints / parameters	5106 / 0 / 413	2421 / 1 / 215
Absorption correction	Analytical	Analytical
Max. and min. transmission	0.950 and 0.866	0.813 and 0.659
Goodness-of-fit on F^2	1.046	1.054
Final *R* indices [$I > 2\sigma(I)$]	*R*1 = 0.0290, w*R*2 = 0.0763	*R*1 = 0.0402, w*R*2 = 0.1048
R indices (all data)	*R*1 = 0.0318, w*R*2 = 0.0781	*R*1 = 0.0437, w*R*2 = 0.1075
Largest diff. peak and hole	0.543 and -0.455 e.$Å^{-3}$	0.993 and -0.425 e.$Å^{-3}$

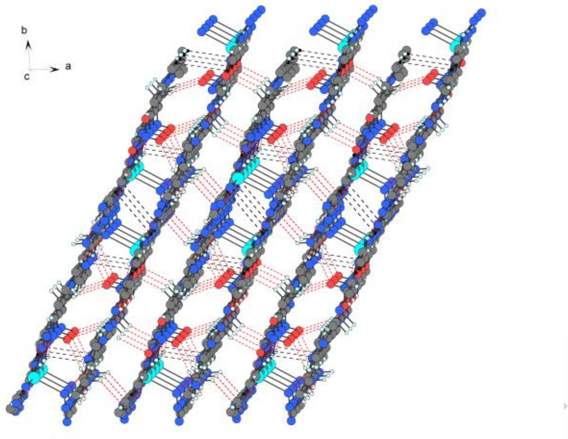

Figure SI1. 3*D* structure of **1** formed by hydrogen bonds (red dashed lines) and *π*–*π* interactions (black dashed lines) between pyridyl rings.

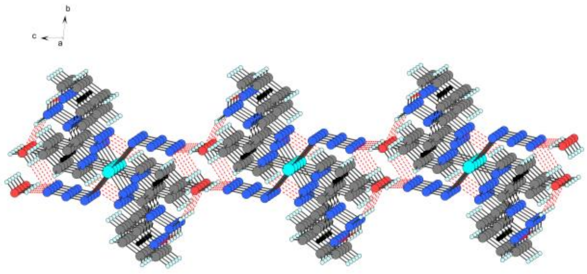

Figure SI2. 2*D* structure of **2** formed by hydrogen bonds (red dashed lines) and *π*–*π* interactions (black dashed lines) between pyridyl rings.

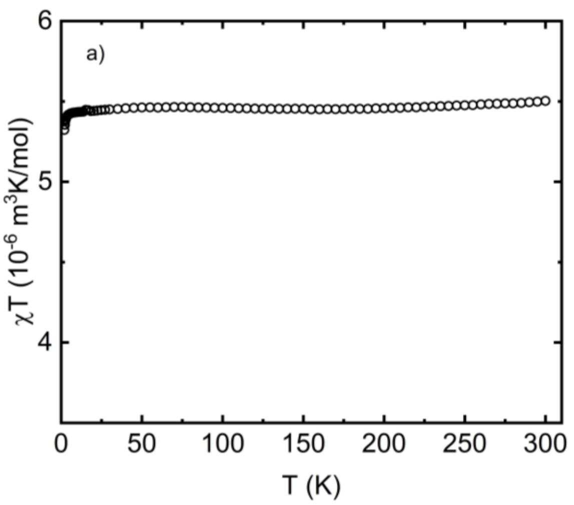

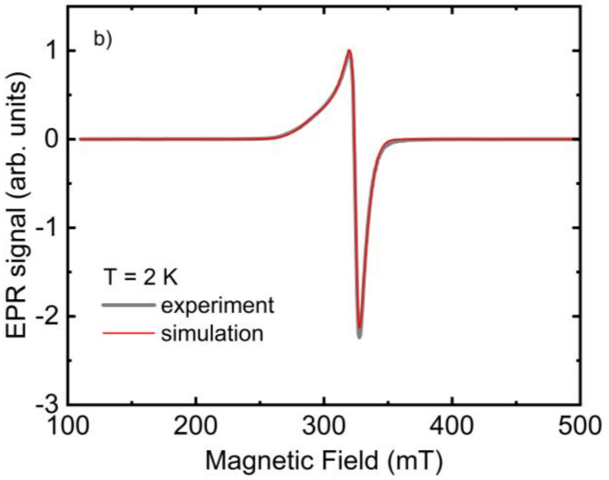

Figure SI3. a) The temperature dependence of the χT of **1**. b) X-band EPR spectra of **1** at 2 K (grey solid line) including a simulation with [g_x, g_y, g_z] = [2.056, 2.066, 2.265] and anisotropic line broadening [ΔB_x, ΔB_y, ΔB_z] = [530, 118, 1182] MHz (red solid line).

Analysis of the Slow Magnetic Relaxation

The extraction of the relaxation times was performed using a modified Debye model accounting for two independent relaxation processes given by the formula

$$\chi(\omega) = \chi_S + \sum_{k=1}^{K} \frac{\chi_{Tk} - \chi_{Tk-1}}{1 + (i\omega\tau_k)^{1-\alpha_k}} \qquad \text{(Equation SI1)}$$

where χ_S is the overall adiabatic susceptibility, χ_{Tk} is the isothermal susceptibility, $\omega = 2\pi f$ is the angular frequency, τ_k is the relaxation time for *k*-th relaxation process, α_k describes the distribution of relaxation times of k-th relaxation process, and *K* defines the number of relaxation channels. The real and the imaginary parts of Equation SI1 were extracted by a fitting script in MATLAB and fitted to the experimental data of χ' and χ'' simultaneously.

A complete formula that would include all possible relaxation mechanisms (field-dependent Raman described by Brons-Van Vleck model, direct, Orbach, and quantum tunnelling process) coexisting in one relaxation channel can be written as

$$\tau^{-1} = \tau_{BVV}^{-1} + \tau_D^{-1} + \tau_{ORB}^{-1} + \tau_{QTM}^{-1} = d\frac{1+eB^2}{1+fB^2} + ATB^4 + \tau_0^{-1}e^{-\frac{U_{eff}}{k_BT}} + \frac{D_1}{1+D_2B^2}$$

(Equation SI2)

where *d* represents a zero-field Raman process $\tau_{ZF}^{-1} \equiv \tau_R^{-1} = CT^n$, with characteristic constants *A*, *C*, D_1, D_2, *e*, *f*, τ_0, and U_{eff} for each process.

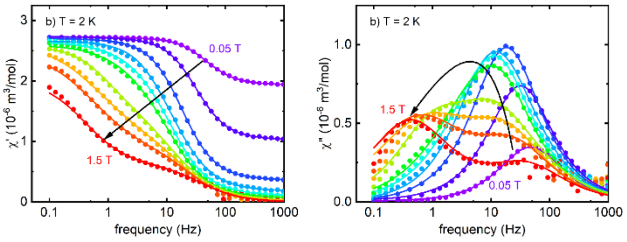

Figure SI4. Frequency dependences of χ' (a) and χ'' (b) of **1** obtained at 2 K at various *dc* magnetic fields (symbols), including the fits (solid lines of corresponding colour) using the modified Debye model Equation SI1 with two relaxation channels at higher fields included.

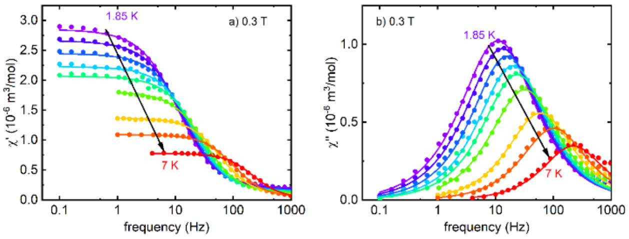

Figure SI5. Frequency dependences of χ' (a) and χ'' (b) of **1** obtained at *dc* field of 0.3 T at various temperatures (symbols), including the fits (solid lines of corresponding colour) using the modified Debye model Equation SI1.

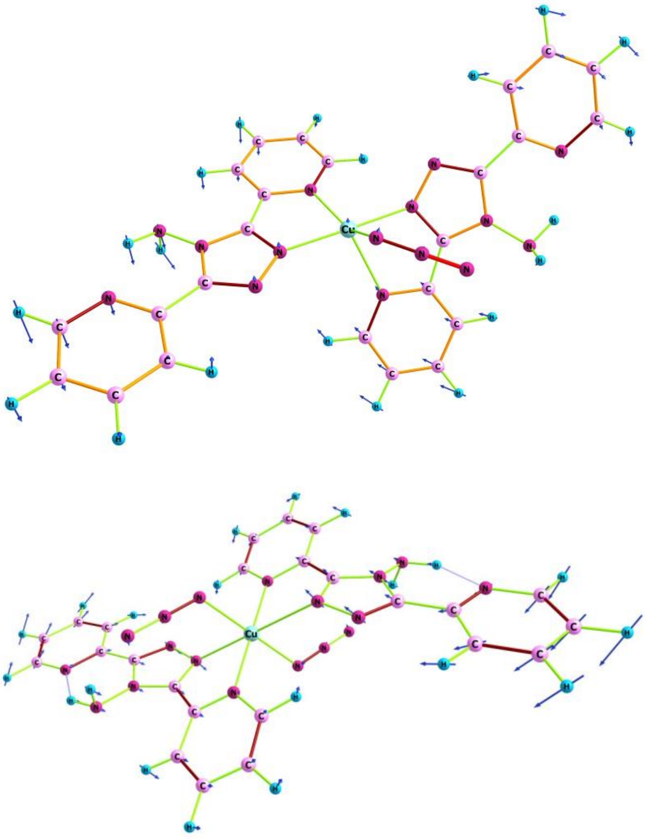

Figure SI6. Intramolecular vibrational modes with energy 16.99 cm^{-1} for **1** (upper panel) and 29.14 cm^{-1} for **2** (lower panel) were calculated by ORCA. Geometry was optimized in the gas phase. Blue arrows show the displacement

vectors, plotted using Chemcraft - a graphical software for visualization of quantum chemistry computations. https://www.chemcraftprog.com.

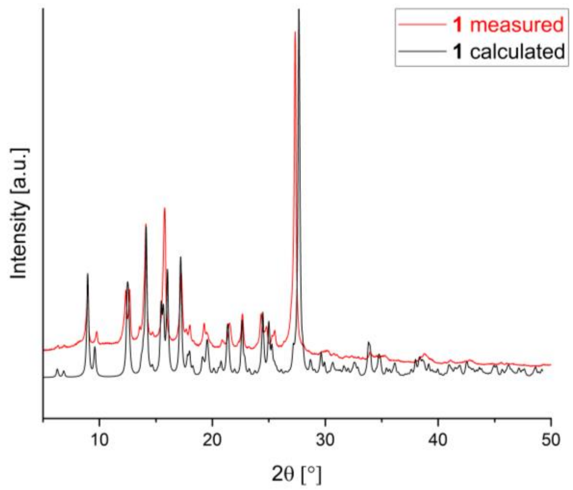

Figure SI7. A comparison of the measured diffraction pattern of **1** with the diffraction pattern calculated from the crysal structure of **1**.

Appendix A

Deposition Numbers CCDC 2408573 and 2408574 contain the supplementary crystallographic data for this paper. These data are provided free of charge by the joint Cambridge Crystallographic Data Centre and Fachinformationszentrum Karlsruhe "http://www.ccdc.cam.ac.uk/structures" Access Structures service.

WILEY - VCH

WILEY-VCH
